# Synthesis of (Bi_1−*x*_Sb_*x*_)_2_S_3_ solid solutions *via* thermal decomposition of bismuth and antimony piperidinedithiocarbamates†

**DOI:** 10.1039/c9ra01127g

**Published:** 2019-05-21

**Authors:** Walter N. Kun, Paul D. McNaughter, Linda D. Nyamen, Ben F. Spencer, Paul O'Brien, Peter T. Ndifon, Neerish Revaprasadu

**Affiliations:** Department of Inorganic Chemistry, University of Yaoundé I. P. O. Box 812 Yaoundé Cameroon; School of Materials, University of Manchester Oxford Road Manchester M13 9PL UK; Schools of Chemistry and Materials, University of Manchester Oxford Road Manchester M13 9PL UK; Department of Chemistry, University of Zululand Private Bag X1001, Kwa-Dlangezwa 3886 South Africa RevaprasaduN@unizulu.ac.za

## Abstract

The synthesis of the complete range of (Bi_1−*x*_Sb_*x*_)_2_S_3_ solid solutions, where 0 ≤ *x* ≤ 1, by the variation of the mole ratio of bismuth and antimony piperidine dithiocarbamate complexes is reported. There was a near linear expansion of *a* and *c* lattice parameters as the mole ratio of the antimony precursor was increased. The composition of the particles directionally followed the amount of precursor ratio used. When the composition of particles was compared to cell parameters, a slight deviation from Vegard's law was observed with a corresponding contraction of the *b* parameter and an approximately 3.5% reduction of the lattice volume. The nanorods obtained showed aspect ratios that depend on the composition of the material. The Bi and Sb rich materials had high aspect ratios of 16.58 and 16.58 respectively with a minimum aspect ratio of 2.58 observed for *x* = 0.50.

## Abstract 2

A full range of (Bi_1−*x*_Sb_*x*_)_2_S_3_ solid solutions from bismuth and antimony piperidine dithiocarbamate complexes is reported.

## Graphical abstract

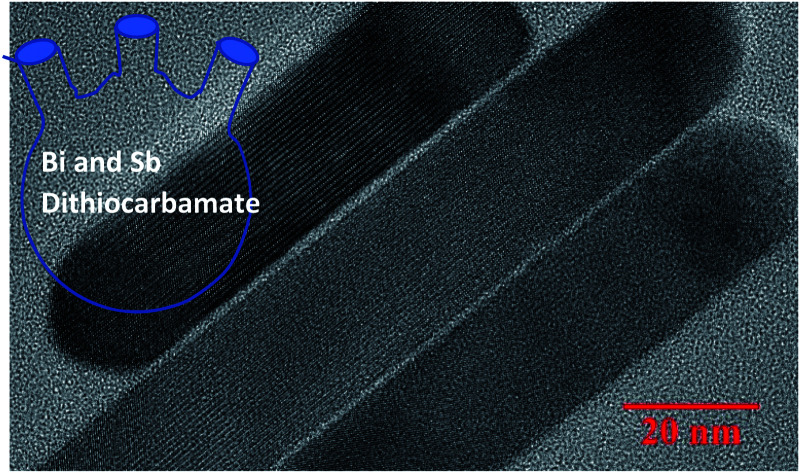

## Introduction

The composition, size, shape and surface topography play important roles in the performance of electronic, optoelectronic, and energy devices.^1^ The incorporation of a foreign atom or ion into a host crystal lattice of a semiconductor material can introduce new functional properties.^2–4^ Group V–VI compounds have attracted much attention due to their earth abundance and environmentally friendly nature. They display many important properties suitable for optoelectronic applications.^1,5–8^ Bi_2_S_3_ has a direct band gap between 1.30 eV and 1.70 eV close to the optimal band gap for terrestrial solar cell energy conversion and a high energy conversion efficiency (≥10^5^ cm^−1^) and as such, is widely used for photovoltaic materials and photodiode arrays.^9–13^ Sb_2_S_3_ has an energy band gap between 1.78 eV and 2.50 eV covering the visible and the near infrared region of the electromagnetic spectrum and shows good photovoltaic properties.^14,15^ It has found use in thermoelectric devices, write once and read many (WORM) optical storage devices, IR region television cameras, infrared spectroscopy and in lithium/sodium ion batteries.^16–22^ solar cells based on Sb_2_S_3_ films have been fabricated with power conversion efficiency of 4.3%.^23,24^

Despite the significant difference in their sizes Bi and Sb form a full range solid solution series between stibnite and bismuthinite.^25,26^ The formation of a solid solution between the two compounds is due to their similarity in charge, ability to crystallize in the same orthorhombic lattice with space group *Pnma*, having typical lattice parameters of *a* = 11.2690 Å, *b* = 3.9717 Å and *c* = 11.1290 Å for bismuthinite and *a* = 11.2990 Å, *b* = 3.8313 Å and *c* = 11.2270 Å for stibnite.^27,28^ Their orthorhombic unit cell volume differs by 3.5%.^29–32^ Analysis on various stibnite and bismuthinite samples from various localities show that replacement of Sb with Bi goes up to 55 moles% giving a limiting mixability range of (Bi_0.45_Sb_0.55_)_2_S_3_ in naturally occurring Bi_2_S_3_–Sb_2_S_3_ solid solution.^25,32,33^ This paucity of representation covering the whole solid solution range in natural samples is attributed to the different geological conditions under which bismuthinite and stibnite are deposited in nature.^25,34^ Kyono *et al.* synthesised a full range (BiSb)_2_S_3_ solid solution series with a nearly statistical substitution of Sb for bismuth by heating Bi_2_S_3_ and Sb_2_S_3_ at 800 to 1000 °C.^30^ However, their method provided no control over stoichiometry as four samples with the same composition range were obtained from a starting mixture with the same Bi_2_S_3_ : Sb_2_S_3_ molar ratio. This observation of large deviations from linear trends in the lattice constants was in contradiction to earlier work by Nayak *et al.* that showed good agreement with Vegard's law on the entire solid solution range by depositing thin films of the solid solution by a dip-dry method.^10^ Colloidal synthesis of nanostructures in surface passivating agents has proven to be an efficient route as it provides easy control over size and shape.^35,36^ Wang *et al.* used a dual precursor source route to synthesize a full range solid solution of (Bi_1−*x*_Sb_*x*_)_2_S_3_ with aspect ratios that depended on their compositions.^1^ Patra *et al.* did similar work using diethyldithiocarbamate complexes in oleylamine and thiol.^4^ However, they did not investigate the influence of the Sb substitution on the lattice constant.^37^ Khan *et al.* prepared the entire range of (SnS_1−*x*_Se_*x*_) from bis(selonobenzoato)dibutyltin(iv) and bis(thiobenzoato)-dibutyltin(iv) complexes by colloidal and melt methods and showed that the colloidal method provided superior control over composition, though both methods showed compositional dependence in the variation of the lattice parameters.^38^ In our earlier work, we showed that addition of a small amount of dodecanethiol was efficient in directing the shapes of Bi_2_S_3_ rods from single source precursors by thermal decomposition of bismuth dithiocarbamate complexes in high boiling point coordination solvents.^39^

This paper examines the effect of substituting antimony for bismuth on the structure of the bismuthinite–stibnite solid solution prepared by bismuth piperidine and antimony piperidine dithiocarbamate complexes.

## Experimental section

Bismuth trichloride (98%, Sigma-Aldrich), antimony trichloride (99%, Sigma-Aldrich), carbon disulphide (99.9%, Sigma-Aldrich), piperidine (99.5% Sigma-Aldrich), oleylamine (98% Sigma-Aldrich), 1-dodecanethiol (98% Sigma-Aldrich), ethanol (99.8% Sigma-Aldrich), chloroform (99.8% Sigma-Aldrich), chloroform-d (99.8% Sigma-Aldrich), and sodium hydroxide (97% Fisher Scientific) were used as supplied without further purification.

### Synthesis of the precursors

#### Preparation sodium piperidine dithiocarbamate (1)

The synthesis of (1) followed previously reported procedure with modifications.^39^ In a typical synthesis, carbon disulfide (0.1 mol, 6.0 mL) was added to an equimolar mixture of sodium hydroxide (0.1 mol, 4.0 g) and piperidine (0.1 mol, 9.9 mL) cooled to 0 °C. After 15 min, the white precipitate formed was filtered, dried in air and recrystallised from acetone/petroleum ether. Na(S_2_CPip): yield: 92%/mp 295 °C. Significant IR bands: *ν* = 3377 (O–H), 964 (C=S), 1468 cm^−1^ (C=N); elemental analysis (%) for C_6_H_14_NS_2_O_2_Na: C 32.86, H 6.43, N 6.39, Na 10.48, S 29.24; found: C 33.05, H 6.34, N 6.6.33, Na 10.66, S 28.88.

#### Preparation of tris(piperidindithiocarbamato)bismuth(iii) (2)

The synthesis of (2) followed our previous procedure with modifications.^37^ BiCl_3_ (5.0 mmol, 1.58 g) was suspended in ethanol (15.0 mL), and added dropwise to a solution of the piperidine dithiocarbamate ligand (15.0 mmol, 2.75 g) in ethanol (25.0 mL) followed by stirring for 1 h. The yellow precipitate formed was collected by filtration and recrystallized from chloroform. Bi(PipDtc)_3_·H_2_O: yield 89% mp 230 °C. Significant IR bands: *ν* = 3477 (O–H), 967 (C=S), 1468 cm^−1^ (C=N); elemental analysis (%) for C_18_H_32_N_3_OS_6_Bi; calc; C 30.54, H 4.56, N 5.94, S 27.18, Bi 29.52. Found; C 30.99, H 4.28, N 5.98, S 26.80, Bi 29.39.

#### Preparation of tris(piperidindithiocarbamato)antimony(iii) (3)

The procedure for (3) was the same as (2) with some modifications. SbCl_3_ (5.0 mmol, 1.14 g) was suspended in ethanol (15.0 mL) and added dropwise to a solution of the piperidine dithiocarbamate ligand (15.0 mmol, 2.75 g) in ethanol (25.0 mL). The resultant solution was stirred for 1 h. The pale-yellow precipitate formed was collected by filtration and recrystallized from chloroform.

Sb(PipDtc)_3_·3H_2_O: yield 82% mp 239 °C. Significant IR bands: *ν* = 3377 (O–H), 967 (C=S), 1476 cm^−1^ (C=N); elemental analysis (%) for C_18_H_36_N_3_O_3_S_6_Sb; calc; C 32.92, H 5.53, N 6.40, S 29.30, Sb 18.54. Found; C 32.72, H 5.84, N 6.47, S 30.14, Sb 19.15.

### Synthesis of (Bi_*x*_Sb_1−*x*_)_2_S_3_ solid solutions

(Bi_*x*_Sb_1−*x*_)_2_S_3_ solid solutions were prepared by variation of the mole ratio of (2) and (3). In a typical experiment, a mixture of (2) and (3) totalling 0.29 mmol was dispersed in a mixture of oleylamine (4.0 mL) and 1-dodecanethiol (0.2 mL). This was injected into 8.0 mL of hot oleylamine (230 °C) under N_2_. After 30 min the reaction was quenched, and the ensuing black precipitate washed three times with ethanol (12.0 mL), centrifuged (11 000 rpm) and dispersed in toluene (5.0 mL).

### Instrumentation

Fourier transform infrared spectroscopy was performed using a Thermo Scientific Nicolet iS5 instrument (4000–400 cm^−1^, resolution 4 cm^−1^). Optical measurements were performed on a Shimadzu UV-1800 spectrophotometer. Elemental analysis was performed with a Thermo Flash 2000 and Carlo Erba EA 1108 elemental analysers (Department of Chemistry University of Manchester). Melting points were recorded on a Stuart SMP10 Melting point apparatus. Thermogravimetric analysis was performed on a Seiko SSC5200/S220TG/DTA model, at a heating rate of 10 °C min^−1^ from 30 °C to 600 °C, under nitrogen.

XRD patterns of the thin films were collected on a PANalytical X'Pert PRO powder diffractometer (Material Science University of Manchester) with a Cu Kα radiation source (*λ* = 1.5406 Å). The samples were mounted flat and scanned over the 2*θ* range of 10–70° in a step size of 0.05.

X-ray photoelectron spectroscopy (XPS) was performed using an Axis Ultra Hybrid (Kratos Analytical, United Kingdom) using 10 mA emission (150 W) of monochromated Al Kα radiation (1486.6 eV). Samples were pressed onto carbon tape, and a charge neutraliser was used to replenish electrons at the surface and remove the effects of differential charging under the X-ray beam. High resolution spectra were collected using an electron energy analyser pass energy of 20 eV and survey spectra with 80 eV pass energy.

X-ray photoelectron spectroscopy (XPS) data were analysed using CASAXPS (www.casaxps.com): the binding energy scales were calibrated using the principle C 1s peak associated with hydrocarbon at 284.8 eV, Shirley backgrounds were fitted where appropriate, and atomic concentrations were calculated using relative sensitivity factors incorporating the photoionization cross section for each core electron orbital, as well as the transmission function of the electron energy analyser. Peak fitting using Voigt-approximation Gaussian–Lorentzian products was performed to obtain binding energy positions for chemical species determination.

Transmission electron microscope (TEM) images, high resolution transmission electron microscope (HRTEM) images, selected area electron diffraction (SAED) patterns and energy dispersive X-ray spectroscopy (EDS) spectra were obtained with an FEI Talos F200A microscope (PSI, University of Manchester) equipped with an X-FEG electron source and Super-X SDD EDS detectors. The experiment was performed using an acceleration voltage of 200 kV and a beam current of approximately 5 nA. Images were recorded with a FEI CETA 4k × 4k CMOS camera. Single crystal X-ray data were collected on a dual source Rigaku FR-X rotating anode diffractometer using Cu Kα wavelength at 150 K and reduced using CrysAlisPro 171.39.30c. Absorption correction was performed using empirical methods (SCALE3 ABSPACK) based upon symmetry-equivalent reflections combined with measurements at different azimuthal angles. The structure was solved and refined using Shelx-2016 implemented through Olex2 v1.2.9.2,3.

## Results and discussion

### Characterization of the precursors

The reaction of piperidine dithiocarbamate with BiCl_3_ and SbCl_3_ gave tris(piperidinedithiocarbamato)bismuth(iii) (Bi(S_2_CPip)_3_) (2) and tris(piperidinedithiocarbamato)antimony(iii) (Sb(S_2_CPip)_3_) (3) respectively. The presence of the dithiocarbamate moiety in the two complexes was shown by the characteristic thioureide band *ν*(C=N) around the 1450–1500 cm^−1^ region and the *ν*(C=S) band around the 960–1000 cm^−1^. These bands appeared shifted to higher frequencies in the spectrum of the corresponding free ligand. The bidentate nature of the coordination of the dithiocarbamate ligand was shown by the band around 960–1000 cm^−1^ which appeared unsplit.^40^ Broad bands around 3300–3500 cm^−1^ in the spectra of the ligands as well as the antimony piperidine complex is due to the presence of moisture in the compounds.

Single crystals of complex (3) were grown in chloroform/ethanol mixture, and their X-ray crystal structure was determined at 150 K. The low temperature structure of tris(piperidinedithiocarbamato)antimony(iii) (Sb(S_2_CPip)_3_), crystallizes into a six coordinate Sb complex surrounded by three piperidinedithiocarbamato groups bonded through S donor atoms. There are three short Sb–S distances of ∼2.53 Å and three long Sb–S distances of ∼2.9–3.0 Å. There is a stereochemically active lone pair on the Sb atom such that the (seven) steric groups (6 S donors and 1 lone pair) occupy the vertices of an ‘elongated triangular pyramid’ in which there are parallel triangular sets of S donors with the lone pair pointed normal to the planes of 3 S atoms (the short Sb–S and the long Sb–S). A similar structure was reported by Liu and Tiekink^41^ at a much higher temperature of 223 K. The three short Sb–S bond length was found to be ∼2.52 Å and that of the three long Sb–S bonds were 2.86 Å, which favourably compares to those of complex (3). The mean interchelate S–Sb–S bond angles of 93.60 of complex (3) is also comparable to 92.32 of the reported structure. The crystal structure, some selected bond lengths together with the crystallographic data and structural refinement parameters for complex (3) are shown in ESI 2, 3 and 4 respectively.†

**Fig. 1:**
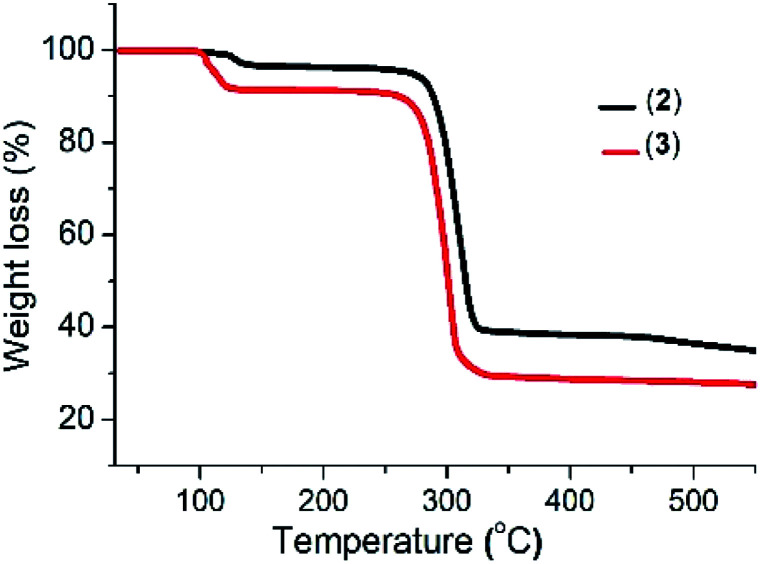
Thermogram of complexes (2) and (3).

The thermogram of complex (2) shows a three-step decomposition pattern. The first mass loss of 3.05% (2.56% calculated) at 128 °C corresponds to the loss of H_2_O molecules. The second mass loss of 59.47% (58.56% calculated) at 281 °C corresponds to the loss of the organic moiety and sulfur, while the third mass loss of 10.41% (11.09% calculated) at 464 °C is attributed to the additional loss of sulfur with the formation of a final residue of 33.67% (35.9% calculated) corresponding to Bi_2_S_3_. Complex (3) shows a two-step decomposition pattern, with the first mass loses of 8.68% (8.14% calculated) at 100 °C corresponding to the loss of the three water molecules and a second mass loss of 70.49% (72.44% calculated) attributed to the loss of the organic moiety and sulfur with the formation of a residue of 26.16% (25.56% calculated) which corresponds to Sb_2_S_3_ (Fig. 1).

### Compositional characterization

**Fig. 2:**
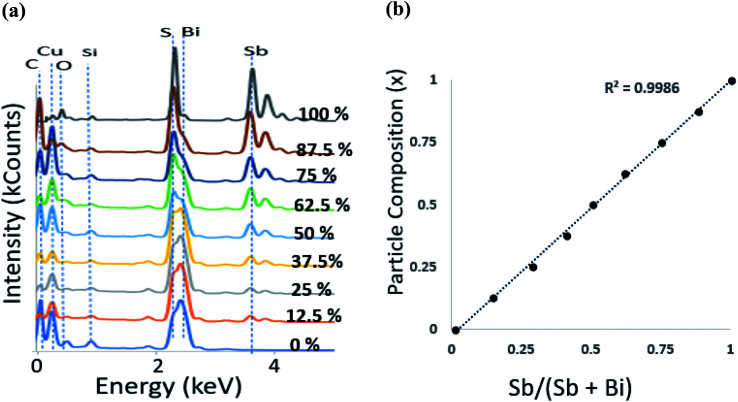
(a) EDS spectra of (Bi_1−*x*_Sb_*x*_)_2_S_3_ nanorods at different Bi : Sb mole ratios (b) particle composition *x* obtained from EDS against precursor mole fraction.

The EDX data (Fig. 2) (where the sampling depth is ≫ the nanorod diameter) shows that the composition of the particles synthesised is in close agreement with the mole fraction of precursors used. This result is unusual with single source precursors as the difference in metal–sulfur bond strengths normally governs the rate of decomposition and consequentially skews the composition to the more reactive metal–sulfur bond. A plot of Sb precursor mole fraction against the proportion of antimony in the particles gave a close to straight line (Fig. 2b) with full details in Table 1. Many of the samples were sulfur rich which is possibly due to the relatively low reaction temperature which prevented evaporation of sulfur, a situation commonly observed in samples prepared at a much higher temperature.^42,43^

**Table 1:** Structural data for Bi_2_S_3_, Sb_2_S_3_ and (Bi_*x*_Sb_1−*x*_)_2_S_3_ solid solution

*X*_Bi_ (%)	*X*_Sb_ (%)	Chemical composition (EDX)	*a* (Å)	*b* (Å)	*c* (Å)	*V* (Å)^3^
100.0	0.0	Bi_1.95_S_3.05_	11.24	3.97	11.13	496.68
87.5	12.5	Sb_0.27_Bi_1.68_S_3.05_	11.24	3.95	11.14	494.32
75.0	25.0	Sb_0.58_Bi_1.34_S_3.08_	11.24	3.93	11.14	492.90
62.5	37.5	Sb_0.79_Bi_1.15_S_3.06_	11.25	3.92	11.16	491.88
50.0	50.0	Sb_0.97_Bi_0.95_S_3.08_	11.26	3.91	11.19	492.00
37.5	67.5	Sb_1.22_Bi_0.75_S_3.04_	11.26	3.87	11.19	487.13
25.0	75.0	Sb_1.49_Bi_0.51_S_3.00_	11.26	3.87	11.21	489.03
12.5	87.5	Sb_1.80_Bi_0.24_S_2.96_	11.27	3.86	11.22	487.23
0.0	100.0	Sb_1.93_S_3.07_	11.27	3.82	11.22	483.22

**Fig. 3:**
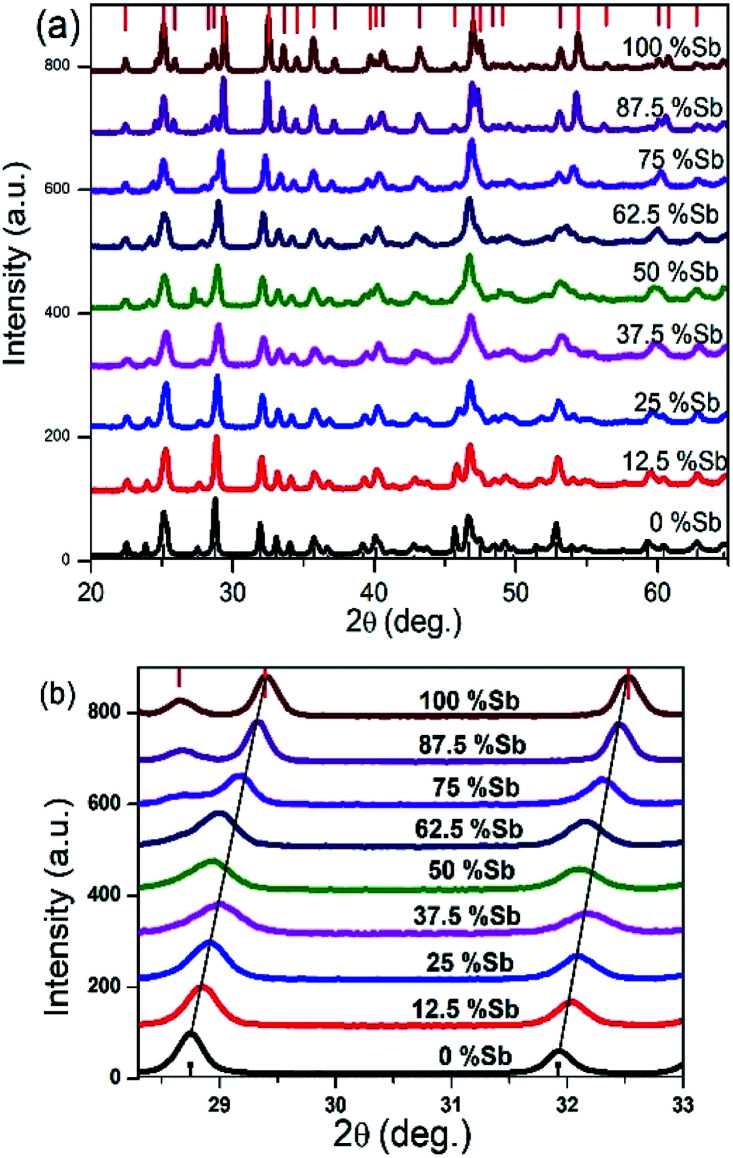
(a) Powder XRD pattern of Bi_2_S_3_ bottom, (Bi_1−*x*_Sb_*x*_)_2_S_3_ and Sb_2_S_3_ top, samples synthesized from different Bi/Sb ratios (b) p-XRD pattern of 2*θ* range 27–33 degree showing shift in peaks.

The X-ray diffraction patterns obtained for all the Bi/Sb ratios correspond well to the orthorhombic crystal system, with peaks for the Bi–Sb–S system falling between previously reported patterns of orthorhombic bismuthinite^26^ (*a* = 11.2690 Å, *b* = 3.9717 Å and *c* = 11.1290 Å) and orthorhombic stibnite^27^ (*a* = 11.2990 Å, *b* = 3.8313 Å and *c* = 11.2270 Å), Fig. 3a. Sb_2_S_3_ and Bi_2_S_3_ both crystallize in the same orthorhombic lattice system with a difference in cell volume of 3.5% due to Sb^3+^ possessing a smaller ionic radius than Bi^3+^. The enlarged portion of the XRD pattern of the samples show a shift in the peak position confirming the successful incorporation of Sb into the Bi_2_S_3_ lattice and the movement through the entire compositional range of the (Bi_1−*x*_Sb_*x*_)_2_S_3_ solid solution, Fig. 3b. A plot of the d-spacing for the (112) plane shows a gradual decrease from Bi_2_S_3_ to the Sb_2_S_3_ end with a percentage difference of 2.26% (ESI 5†).

**Fig. 4:**
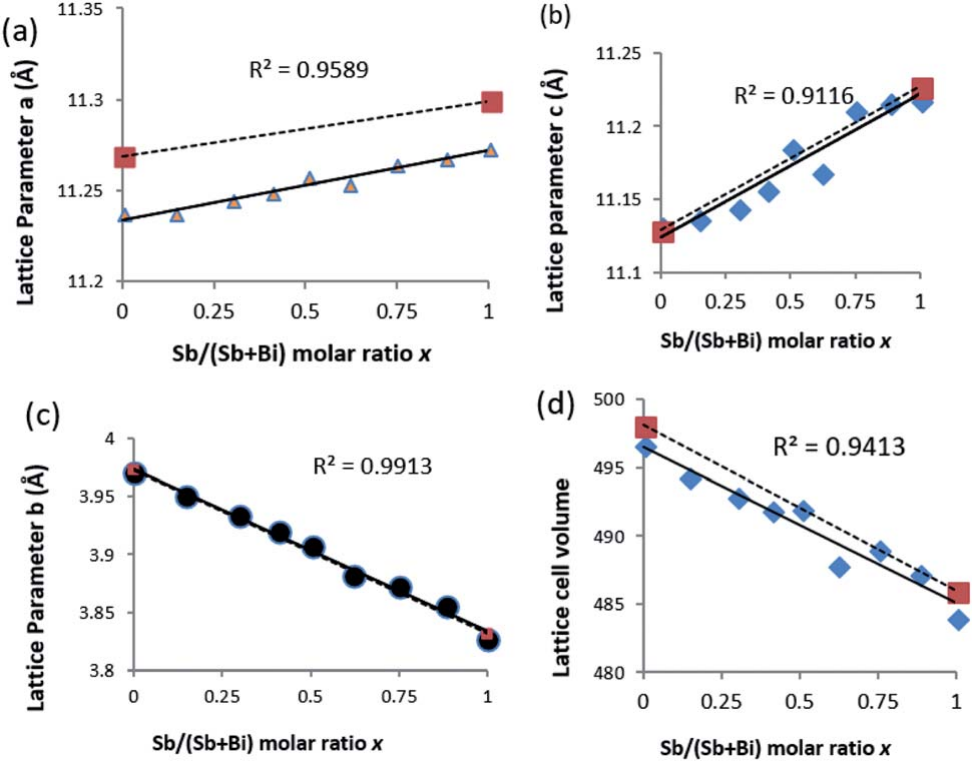
Variation of lattice constants with increasing mole fraction of Sb obtained from EDS. (a) Lattice parameter *a*, (b) lattice parameter *b*, (c) lattice parameter *c* and (d) cell volume. * values represent the reported standard value (dotted lines) for Bi_2_S_3_ and Sb_2_S_3_.

Refinement of the powder XRD data shows that all three axis of the unit cell vary linearly (Fig. 4). Upon increased incorporation of antimony, *a* and *c* increase whereas *b* decreases which is the expected behaviour when moving between Bi_2_S_3_ and Sb_2_S_3_. All three cell parameters show a linear dependency on the amount of antimony in the solid solution which agrees with Vegard's law. The subtle deviations in *a* and *c* from ideal behaviour may be due to the contrasting effect of the stereochemical active lone pair of the 5s^2^ and 6s^2^ electrons of the antimony and bismuth atoms, which is positioned in the *a*–*c* plane of the lattice.^37,44^ With an increased concentration of antimony, there is expansion of the inter-rod space due to the expression of the stereochemically active lone electron pair with a resulting expansion of the *a* and *c* parameters.^30^ However, the *b* axis which is least affected by the stereochemical active lone pair experiences a continuous contraction on Sb substitution, probably due to decrease in the shortest M–S bond as we move from the Bi_2_S_3_ to the Sb_2_S_3_ end.^26^ There is a general shrinkage of the overall cell volume of (Bi_1−*x*_Sb_*x*_)_2_S_3_ as Bi is replaced by Sb (Fig. 4d).

**Fig. 5:**
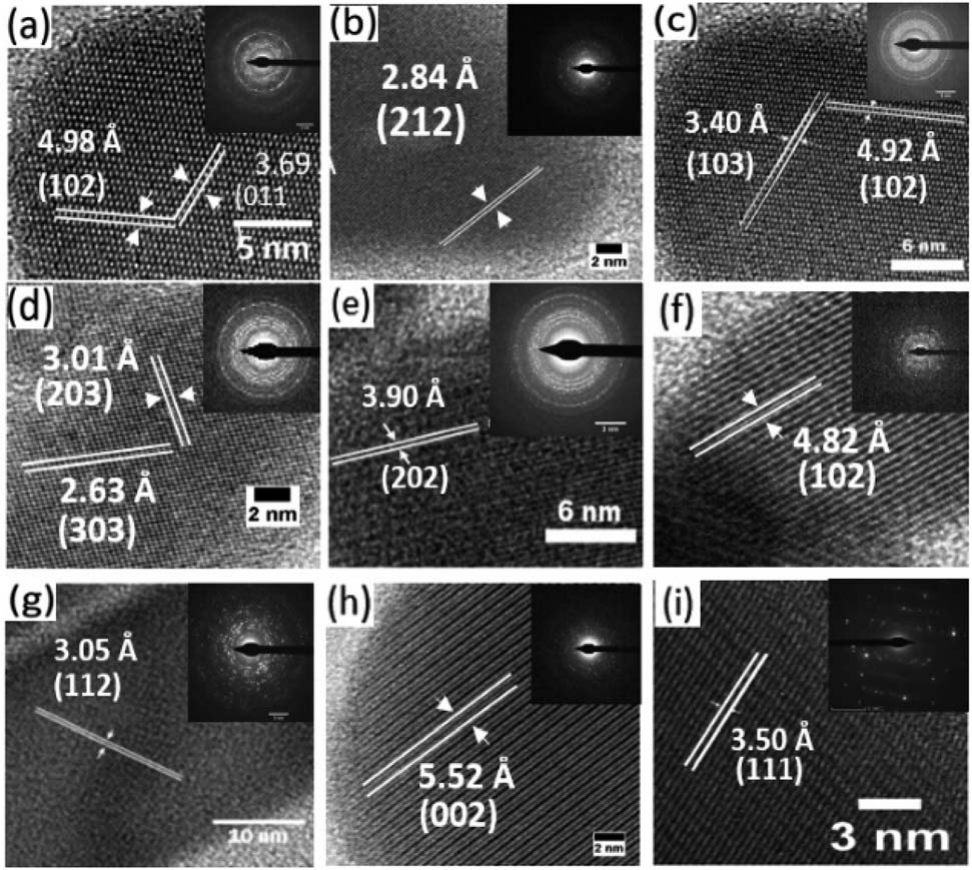
HRTEM images of synthesized nanorods with Sb/(Sb + Bi) mole fraction of (a) 1 : 0, (b) 7 : 1, (c) 3 : 1, (d) 5 : 3, (e) 1 : 1, (f) 3 : 5, (g) 1 : 3, (h) 1 : 7 and (i) 0 : 1. Inset in each image shows the SAED pattern.

The high-resolution transmission electron microscopy (HRTEM) images of the samples together with their selected area electron diffraction (SAED) patterns reveal the formation of highly polycrystalline powders showing two-dimensional lattice fringes (Fig. 4). Measured *d*-spacings of 3.69 and 4.98 Å were obtained for pure Bi_2_S_3_ (Fig. 5a) corresponding to the (011) and (102) planes (SG *Pnma* with *a* = 11.2690 Å, *b* = 3.9717 Å and *c* = 11.1290 Å) while for pure Sb_2_S_3_ (Fig. 5i) a *d*-spacings of 3.50 Å corresponding to the (111) (SG *Pnma* with *a* = 11.2990 Å, *b* = 3.8313 Å and *c* = 11.2270 Å) plane was recorded.

**Fig. 6:**
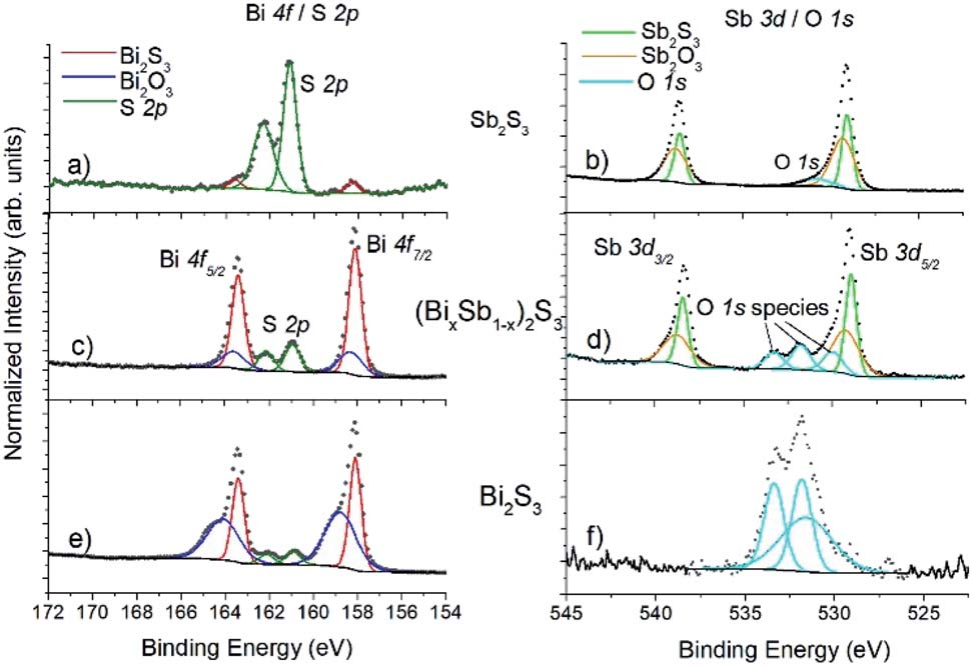
XPS spectra for Sb_2_S_3_ (top panels (a) and (b)), Bi_2_S_3_ (bottom panels (e) and (f)), and (Bi_*x*_Sb_1−*x*_)_2_S_3_ (middle panels (c) and (d)). The Bi 4f and S 2p spectral regions overlap ((a), (c), (e)), and the Sb 3d region overlaps with O 1s ((b), (d), (f)).

XPS is a much more surface sensitive technique than EDX, with sampling depths varying 6.3–9.0 nm for Sb, Bi and S,^45^ which is much less than the nanorod diameter. Bi 4f coincides with the S 2p region, and Sb 3d coincides with O 1s. Fig. 6 shows a pile-up of the Bi 4f/S 2p and Sb 3d/O 1s regions for Bi_2_S_3_, Sb_2_S_3_ and (Bi_1−*x*_Sb_*x*_)_2_S_3_.

In all cases the Bi 4f doublet required two chemical species (two sets of spin–orbit–split doublets) in order to obtain an adequate fit, with positions for the Bi 4f_7/2_ photoelectron peaks at 158.1 eV (associated with Bi_2_S_3_ ^46^ and 158.8 eV (associated with oxidized Bi_2_O_3_.^47^ Likewise, the Sb 3d doublet required two chemical species for adequate fitting, with peak positions for 3d_5/2_ at 259.1 eV (associated with Sb_2_S_3_ ^48^ and 530.1 eV (associated with oxidized Sb_2_O_3_).^49^ Note that O 1s photoelectron peaks are close to the Sb 3d_5/2_ signal, typically with binding energy positions at ∼530.5 eV associated with metal oxides (*i.e.*, BiO_*x*_, SbO_*x*_), ∼532 eV associated with C–O contamination, and ∼533 eV associated with C=O contamination. A variety of (Bi_*x*_Sb_1−*x*_)_2_S_3_ samples were measured, and consistently a peak-fitting model including sulfide and oxide species was required for both Bi and Sb. However, no oxidation was seen for S, only one species for the S 2p doublet was observed for all the samples measured, with the peak position for 2p_3/2_ at ∼161.0 eV associated with sulfide,^46,48^ and in the spectra there is a clear absence of any signal associated with sulfate which is expected in the binding energy region 168–170 eV.^50^ Also, when calculating the atomic ratios of Bi : Sb : S, there is consistently an absence of S as expected for (Bi_1−*x*_Sb_*x*_)_2_S_3_. In Fig. 6 the (Bi_*x*_Sb_1−*x*_)_2_S_3_ sample exhibits a Bi : Sb : S ratio of 3 : 3 : 4 (or 1 : 1 : 1.3, short of the expected 1 : 1 : 1.5). This indicates that there is an absence of sulfur atoms at the surface of the nanorods hence the atomic concentrations (Table 2) are skewed from the bulk measurement by EDX analysis. This also explains the lack of sulfur oxidation while a small amount of Bi and Sb atoms at the surface of the nanorods are susceptible to oxidation. For the range of (Bi_1−*x*_Sb_*x*_)_2_S_3_ nanorod materials measured, the amount of oxidation of Bi and Sb observed varied between 10–40% (with an average value of 20% for Bi and 26% for Sb).

**Table 2:** Relative percentage concentrations of Bi, Sb and S for Bi_2_S_3_, Sb_2_S_3_ and (Bi_1−*x*_Sb_*x*_)_2_S_3_. Bi and Sb are further delineated into sulfide and oxide species, where oxidation occurs between 10–40% of the time (with an average of 20% for Bi and 26% for Sb)

Sb/(Sb + Bi)	Bi–S%	Bi–O%	Total Bi%	Sb–S%	Sb–O%	Total Sb%	S%
0	49.79	18.69	68.49	0.00	0.00	0.00	31.51
0.125	49.11	9.33	58.43	4.44	0.49	4.93	36.64
0.25	45.20	8.61	53.81	7.86	1.56	9.43	36.76
0.375	37.32	8.25	45.57	17.53	1.76	19.29	35.14
0.5	34.86	5.81	40.67	15.55	6.17	21.72	37.60
0.625	27.85	5.32	33.17	20.94	7.39	28.34	38.49
0.75	27.45	3.40	30.85	23.36	6.67	30.03	39.13
0.875	17.09	1.66	18.74	29.75	11.16	40.91	40.35
1	3.56	0.00	3.56	38.20	12.14	50.34	46.10

### Morphological characterization

**Fig. 7:**
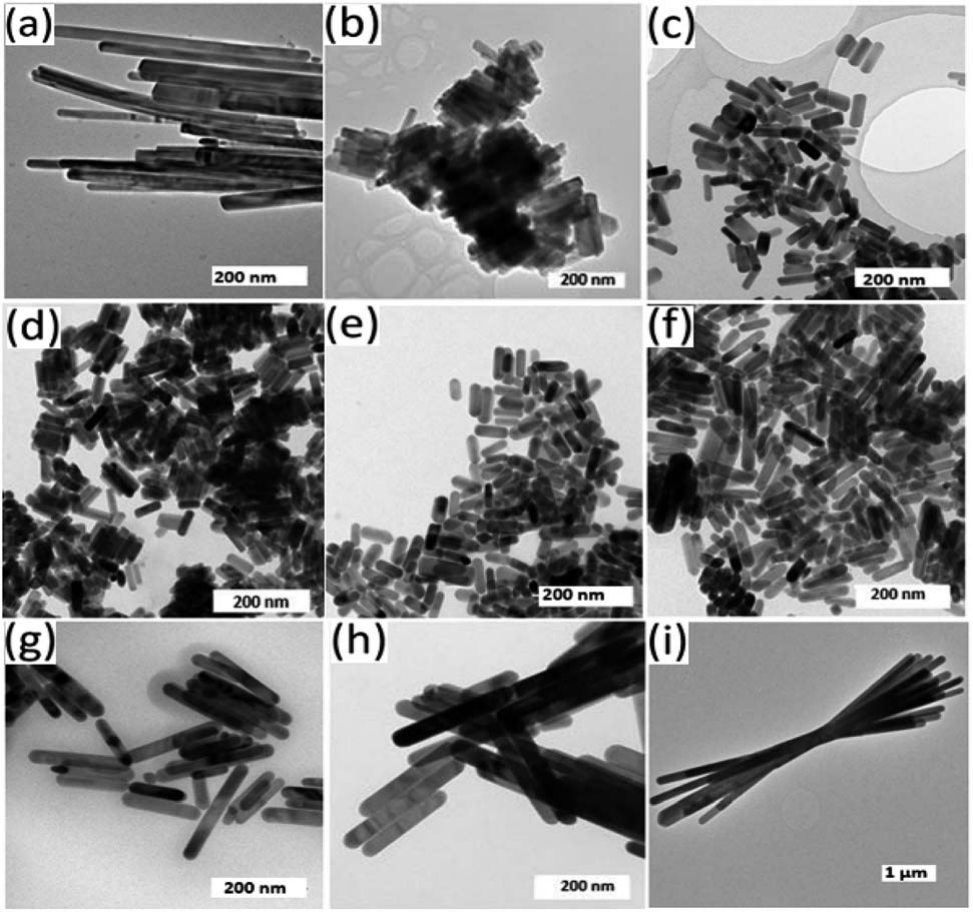
TEM images showing the as synthesized nanorods with Bi : Sb mole ratios of (a) 1 : 0, (b) 7 : 1, (c) 3 : 1, (d) 5 : 3, (e) 1 : 1, (f) 3 : 5, (g) 1 : 3, (h) 1 : 7 and (i) 0 : 1.

**Fig. 8:**
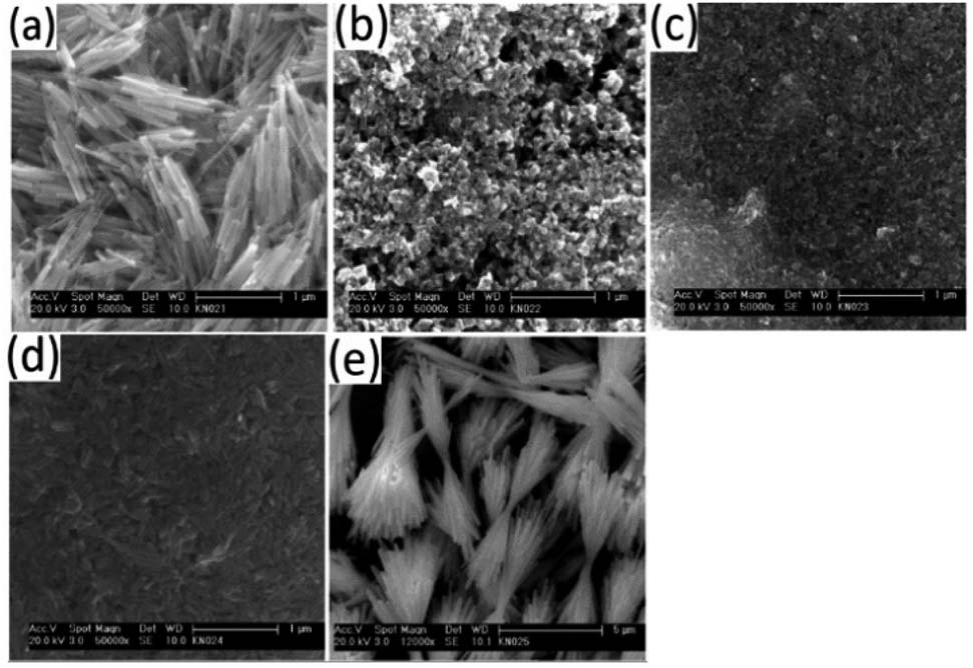
SEM images showing surface scan of films with Bi : Sb mole ratios of (a) 1 : 0, (b) 3 : 1, (c) 1 : 1, (d) 1 : 3, (e) 0 : 1.

TEM analysis on the samples showed the presence of particles with a rod-shaped morphology, Fig. 7a–i. When the bismuth precursor was exclusively used, uniform elongated cylindrical nanorods of Bi_2_S_3_ were obtained however the antimony precursor gave long sheaf-like collections of Sb_2_S_3_ rods in the sub-micrometre range (Fig. 7i and 8e).

There was a conspicuous change in the aspect ratio of the rods as the Bi : Sb precursor mole ratio was varied, Table 3. With a Bi : Sb precursor mole ratio of 7 : 1, there was a considerable reduction in both the length and aspect ratio of the nanorods compared to pure Bi_2_S_3_ until a mole ratio of 1 : 1, which gave an aspect ratio of 2.58 (Table 3). Wang *et al.* prepared (Bi_1−*x*_Sb_*x*_)_2_S_3_ 1-d rods, by reacting bismuth chloride, antimony chloride, sulphur powder, oleylamine and thiols and observed composition dependant aspect ratios.^1^ Sun *et al.* synthesized flower-like architectures Sb_2−*x*_Bi_*x*_S_3_ by solvothermal treatment of bismuth and antimony diethyldithiocarbamate complexes. They proposed a mechanism in which 3-d flowers grow through an epitaxial growth on Sb_2−*x*_Bi_*x*_S_3_ core.^51^ When we increased the precursor mole ratio of Sb beyond 1 : 1, the aspect ratio increased along with the length of the rods. At a mole ratio of 1 : 7 the longest rods of the solid solution were observed. Sb_2_S_3_ has a partial fractal splitting growth habit which often lead to the formation of sheaf-like morphologies.^52^ However, the inclusion of Bi ions alters the growth dynamics by inducing complete splitting growth and consequently changes morphology from very long sheaf-like sub-micrometre Sb_2_S_3_ rods to shorter separate nanorods. The particle size distribution of the as synthesized nanorods are shown in ESI 6.† TEM images of intermediate Bi : Sb ratio to those reported are shown in ESI 7.†

**Table 3:** Dimensions of synthesised nanorods of Bi_2_S_3_, Sb_2_S_3_ and (Bi_1−*x*_Sb_*x*_)_2_S_3_ solid solution

Sb/(Sb + Bi)	Length (nm)	Width (nm)	Aspect ratio
0	474.4 ± 92.62	28.61 ± 12.90	16.58
0.125	104.99 ± 46.38	19.79 ± 9.13	5.31
0.25	65.13 ± 13.52	23.69 ± 6.50	2.75
0.375	57.00 ± 11.12	15.55 ± 4.07	3.66
0.5	60.38 ± 12.60	23.38 ± 4.38	2.58
0.625	25.04 ± 7.37	5.56 ± 1.09	4.51
0.75	142.81 ± 56.58	28.56 ± 5.01	5.00
0.875	386.32 ± 137.43	40.05 ± 11.08	9.65
1	2880.47 ± 550.22	137.09 ± 44.82	21.01

### Optical properties

**Fig. 9:**
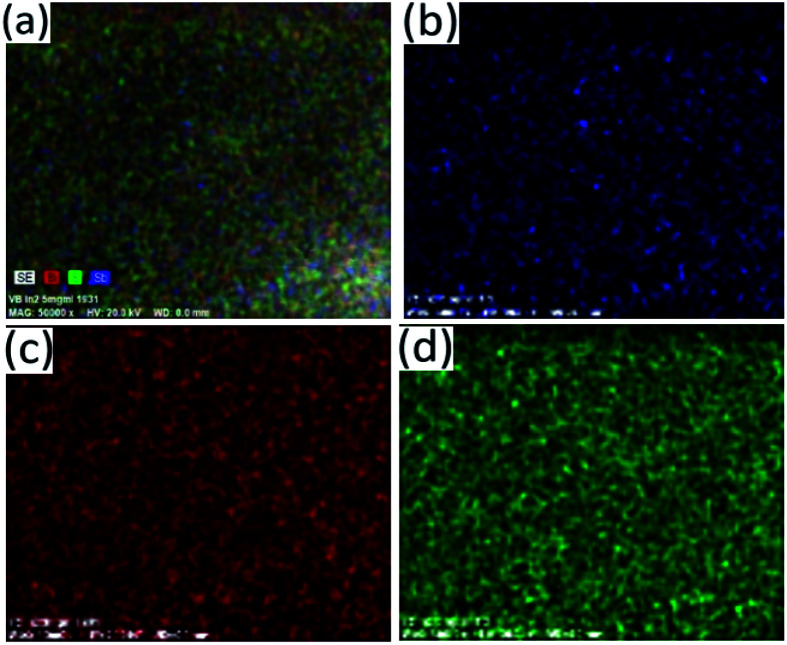
Elemental mapping of the particles synthesized at Bi : Sb mole ratio of 1 : 1 showing distribution of atoms (a) SE, (b) Sb, (c) Bi and (d) S.

Bi_2_S_3_ and Sb_2_S_3_ possess direct band gaps of approximately 1.3 and 1.7 eV respectively, corresponding to 954 and 729 nm. The band gap of the ternary (Bi_1−*x*_Sb_*x*_)_2_S_3_ solid solutions made using different ratios of Bi and Sb should be a linear interpolation of the two parent materials. Fig. 9a shows the UV-visible absorption spectra of the as-prepared ternary (Bi_1−*x*_Sb_*x*_)_2_S_3_ in which a strong broad absorption was seen within the wavelength range of 300–1100 nm. In general, the absorption edge of (Bi_1−*x*_Sb_*x*_)_2_S_3_ nanorods is blue-shifted with the increase of Sb ratio.

**Fig. 10:**
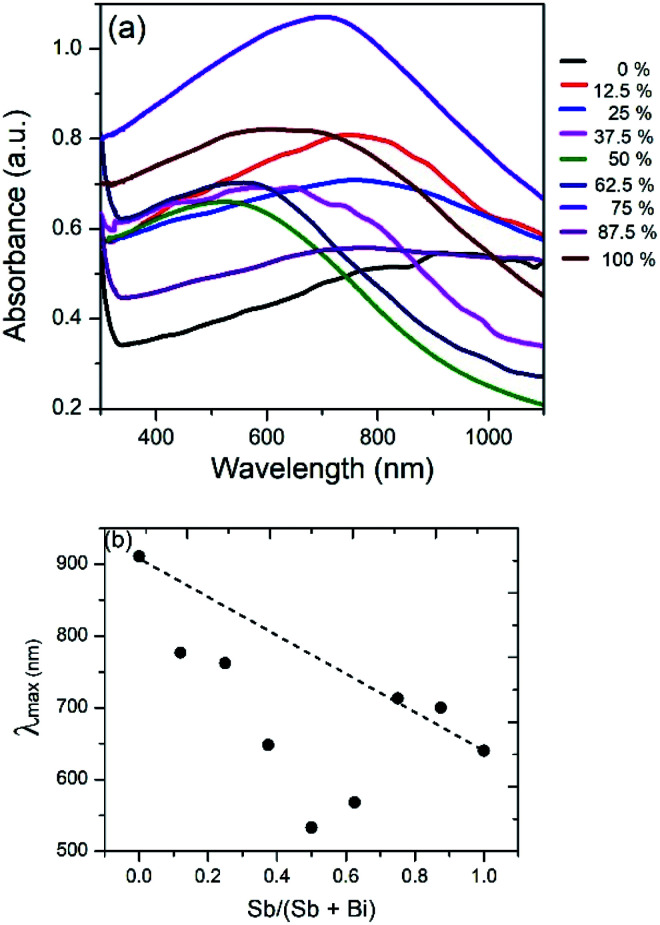
(a) UV/visible absorption spectrum of Bi_2_S_3_, Sb_2_S_3_ and (Bi_1−*x*_Sb_*x*_)_2_S_3_ solid solutions. (b) Plot of absorption maximum against mole fraction of Sb, showing deviation from ideal behaviour.

However, a plot of the absorption maximum against the antimony mole fraction shows a marked deviation from the expected linear behaviour of the band gap of ternary semiconductor materials (Fig. 10). This phenomenon known as band gap bowing is often ascribed to local compositional fluctuations which occur on substitution. The extent of such local atom displacements usually brings about nonlinear dependence on optical properties in ternary materials.^44,53^ O'Brien *et al.* synthesize Bi_2−2*x*_Sb_2*x*_S_3_ solid solutions from solvent less thermolysis of metal xanthate precursors and showed a slight deviation from linearity in the energy band gap on Sb substitution due to stoichiometric variations in the synthesized solid solution.^54^

**Fig. 11:**
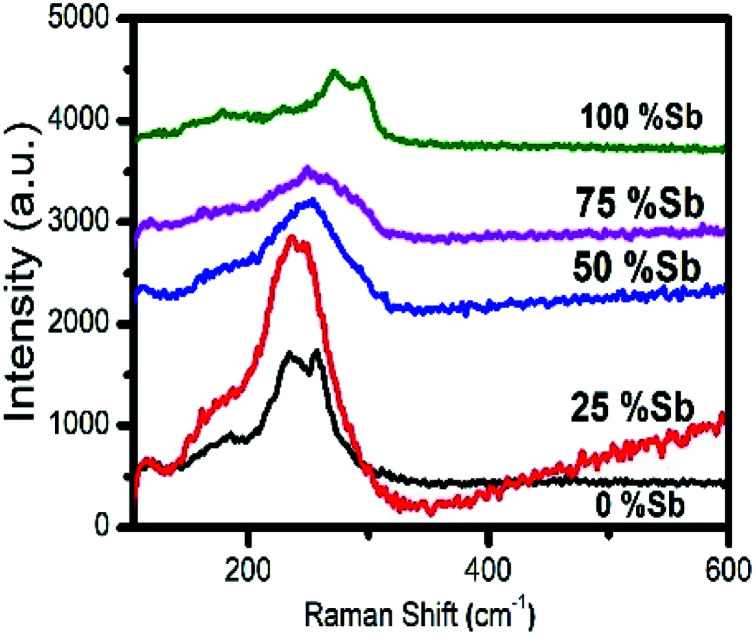
Raman spectra of nanorods of Sb_2_S_3_, Bi_2_S_3_ and (Bi_1−*x*_Sb_*x*_)_2_S_3_ solid solutions.

The Raman spectra of the particles are shown in Fig. 11. In case of pure Bi_2_S_3_ sample, a minor peak was observed at 184 cm^−1^ and two prominent peaks at 236 and 256 cm^−1^, which is in agreement with the previously reported Raman data for Bi_2_S_3_. The minor peak is assigned as A_g_ symmetric bending mode, whereas the dominant peaks (236 and 256 cm^−1^) are A_g_ and B_1g_ anti-symmetric stretching modes, respectively.^55,56^ Similarly, one minor and two major peaks at 186, 272 and 294 cm^−1^ were observed for pure Sb_2_S_3_, which are in good agreement with the previous reports.^57–59^ The peak at 186 cm^−1^ can be assigned to the B_1g_ anti-symmetric S–Sb–S bending modes, whereas the peaks at 272 and 294 cm^−1^ are assigned to the A_g_ and B_1g_ anti-symmetric Sb–S stretching modes, respectively.^56,57^ The solid solutions consisting of 25% antimony show mainly one broad band around 240 cm^−1^, which shift to higher frequencies of 253 and 260 cm^−1^ when the percentage of antimony is increased to 50 and 75% respectively. The shift towards the higher wavenumber is due to lower mass of Sb as compare to Bi and shorter Sb–S bond respectively.^60,61^

## Conclusions

By the thermal decomposition of bismuth and antimony piperidinedithiocarbamates in oleylamine nanorods of the entire compositional range of (Bi_1−*x*_Sb_*x*_)_2_S_3_ of solid solutions have been produced by varying the bismuth and antimony precursor mole fraction. The morphologies of the nanorods depended on their compositions, and aspect ratios that decreased to a minimum of 2.58 with maxima of 16.58 when using just the bismuth precursor and 21.01 when using the antimony precursor.

The XRD peaks at all ratios correspond to the orthorhombic crystals system and fall between those of orthorhombic Bi_2_S_3_ and orthorhombic Sb_2_S_3_. The gradual shift in the peaks position in combination with compositional data from EDX confirms the successful incorporation of antimony into bismuth sulphide which almost adheres to Vegard's law.

## Conflicts of interest

There are no conflicts to declare.

## Supplementary Material

RA-009-C9RA01127G-s001

RA-009-C9RA01127G-s002
