# Supplementary material for: Synthesis of (Bi1−xSbx)2S3 solid solutions via thermal decomposition of bismuth and antimony piperidinedithiocarbamates
Source: RSC Adv. 2019 May 21;9(28):15836–44. doi: 10.1039/c9ra01127g (PMC9064327; doi:10.1039/c9ra01127g)
Supplement: RA-009-C9RA01127G-s001 [file RA-009-C9RA01127G-s001.pdf]

Electronic Supplementary Material (ESI) for RSC Advances.
This journal is © The Royal Society of Chemistry 2019

The synthesis of $(Bi_{1-x}Sb_x)_2S_3$ nanorods ($0 \leq x \leq 1$), using the thermal decomposition of bismuth and antimony piperidinedithiocarbamates

Walter N. Kun, [a,b] Paul D. McNaughter,[b] Linda D. Nyamen,[a] Ben F. Spencer,[c] Paul O'Brien,[b, c] Peter T. Ndifon[a]* and Neerish Revaprasadu[d]*

Supplementary Data

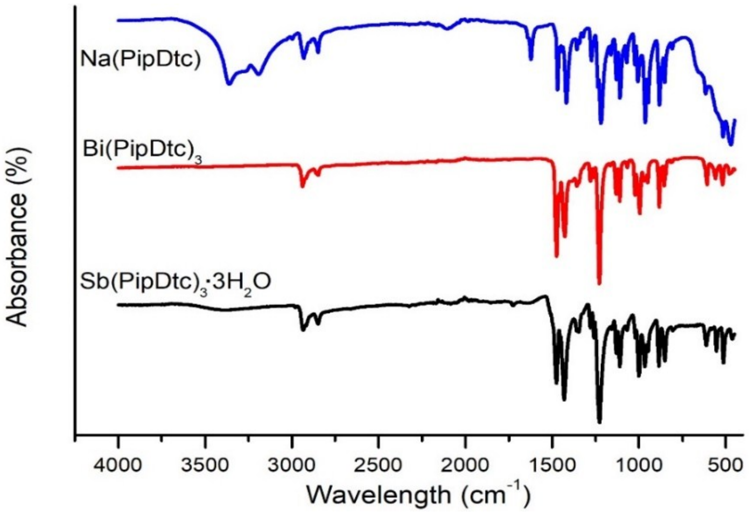

ESI 1 FTIR spectra of ligand and complexes

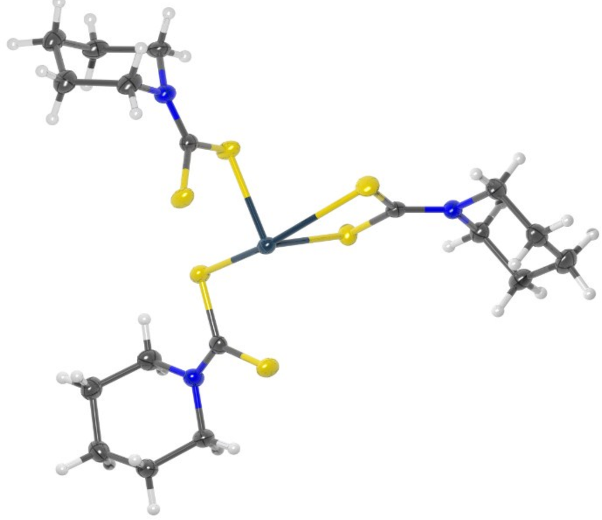

ESI 2 Assymetric unit of tris(piperidinedithiocarbamato)antimony(III) in the crystal (yellow = sulfur, dark grey = antimony, blue = nitrogen, grey = carbon, white = hydrogen) (CCDC 1889653)

ESI 3 Selected bond lengths for ris(piperidinedithiocarbamato)antimony(III)

Bond	Length	Bond	Length
Sb00—S002	2.5330 (4)	S002—Sb00—S003	88.093 (14)
Sb00—S003	2.5358 (4)	S002—Sb00—S004	150.697 (13)
Sb00—S004	2.8918 (5)	S002—Sb00—S006	82.089 (14)
Sb00—S006	2.5337 (4)	S003—Sb00—S004	65.889 (12)
S002—C00E	1.7584 (17)	S006—Sb00—S003	90.215 (14)
S003—C00B	1.7549 (16)	S006—Sb00—S004	84.658 (14)
S004—C00B	1.7027 (16)	S005—C00D—S006	119.59 (10)
S005—C00D	1.6955 (16)	S007—C00E—S002	119.05 (10)
S006—C00D	1.7535 (17)	S004—C00B—S003	118.19 (10)
S007—C00E	1.7007 (17)		

ESI 4 Crystal Data and structural refinement parameters for antimony piperidine dithiocarbamate.

CCDC	1889653
Empirical formula	C18H30N3S6Sb
Formula weight	602.56
Temperature/K	150
Crystal system	monoclinic
Space group	P21/c
a/Å	17.6331(3)
b/Å	11.84939(19)
c/Å	12.12535(19)
α/°	90
β/°	107.4771(17)
γ/°	90
Volume/Å3	2416.54(7)
Z	4
ρcalcg/cm3	1.656
μ/mm-1	13.983
F(000)	1224.0
Crystal size/mm3	0.146 × 0.111 × 0.067
Radiation	CuKα (λ = 1.54184)
2Θ range for data collection/°	5.254 to 136.49
Index ranges	-20 ≤ h ≤ 21, -13 ≤ k ≤ 14, -14 ≤ l ≤ 12
Reflections collected	16485
Independent reflections	4406 [Rint = 0.0187, Rsigma = 0.0170]
Data/restraints/parameters	4406/0/254
Goodness-of-fit on F2	1.056
Final R indexes [I>=2σ (I)]	R1 = 0.0172, wR2 = 0.0422
Final R indexes [all data]	R1 = 0.0179, wR2 = 0.0426
Largest diff. peak/hole / e Å-3	0.31/-0.30

A plot of the d-spacing for the (112) plane shows a gradual decrease from Bi2S3 to the Sb2S3 end with a percentage difference of 2.26%

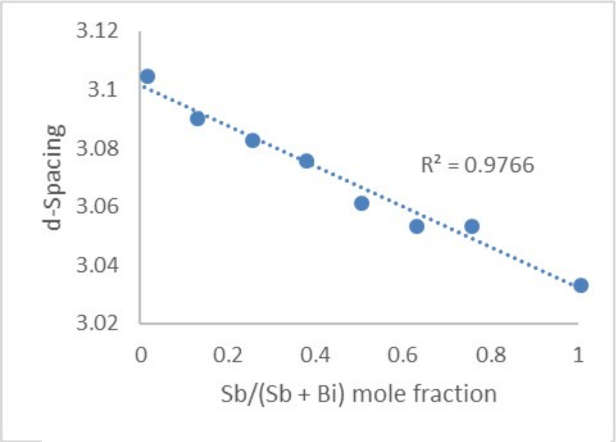

ESI 5 A plot of the d-spacing for the (112) plane

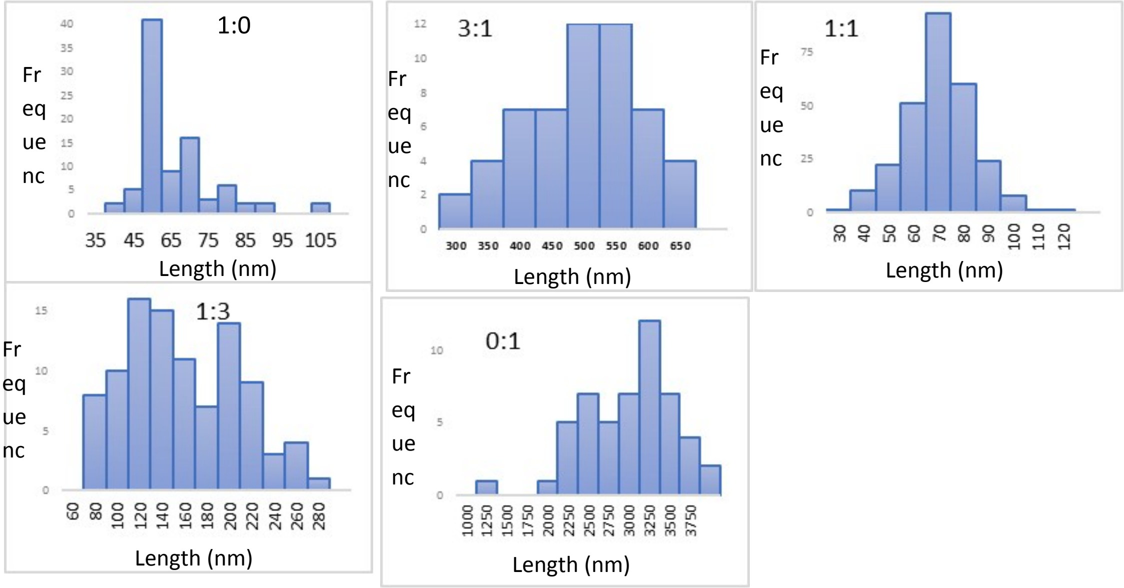

ESI 6. particle size distribution of the as synthesized nanorods against % Sb in nanorods.

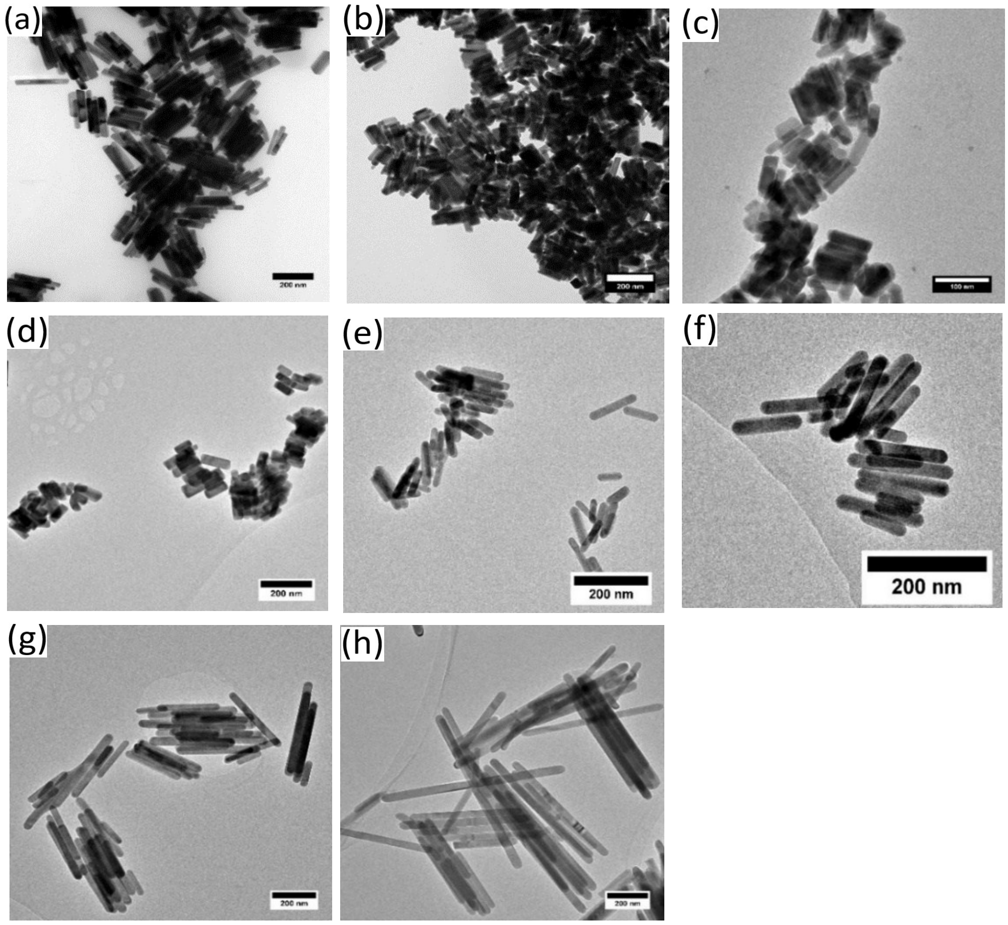

ESI 7. TEM images showing the as synthesized nanorods with Bi:Sb mole ratios of (a)15:1, (b) 13:3 (c) 11:5 d)9:7 (e) 7:9 (f) 5:11 (g)3:13 and (h) 1:15
